# Hypodermic Medication

**Published:** 1885-11

**Authors:** 


### Hypodermic Medication.

In an editorial in the September number of the *St. Louis Medical and Surgical Journal*, the editor wisely remarks that with but few exceptions medicines produce their effect by absorption, regardless of the locality of administration. Experimention long since demonstrated that remedies given subcutaneously and in smaller doses act much more rapidly than when introduced by the stomach, and a knowledge of this fact enables the practitioner to deal successfully with many conditions otherwise uncontrollable. The advantages afforded through this instrumentality are not as freely grasped as would naturally be supposed, physicians generally limiting such medication to the injection of morphia, mainly for the relief of neuralgia. The literature of the subject has been insufficiently studied, and the average practitioner has consequently remained satisfied with the stereotyped methods of medicating. Perhaps, too, the disinclination of the physician to inflict even the minimum of pain incident to the introduction of the needle, coupled with the aversion of most patients to any procedure, even remotely allied to surgery, have operated as deterents to the general adoption of hypodermic medication.

The Onion as an Antidote to Stings and Bites.—We clip the following two items from the *Medical Age:*

"An old woodsman of Australia, who used to catch snakes for pastime, says that a raw onion bruised and applid as soon as possible of all venomous serpents of that country, except the death adder, which he admits is so poisonous, and its poison so quick in acting, that there is no known remedy for it. That the onion is a speific for the sting of poisonous insects of all kinds has long been known to the writer of this paragrapn, who, when a boy, invariable carried one on expeditions with his companions, against hornets' nests, etc. It was found that the application of onion juice would instantly allay the pain caused by the stinging of hornets, yellow-jackets, wasps, etc."

